# Development and the Effect of Weather and Mineral Fertilization on Grain Yield and Stability of Winter Wheat following Alfalfa—Analysis of Long-Term Field Trial

**DOI:** 10.3390/plants12061392

**Published:** 2023-03-21

**Authors:** Lukáš Hlisnikovský, Ladislav Menšík, Eva Kunzová

**Affiliations:** Department of Nutrition Management, Crop Research Institute, Drnovská 507, Ruzyně, 161 01 Prague, Czech Republic

**Keywords:** *Triticum aestivum* L., *Medicago sativa* L., legumes, temperature, precipitation, yield, nitrogen, weather and yield variability, response models

## Abstract

Within the framework of a long-term experiment, established in 1955, we evaluated the development and effects of weather and mineral fertilization (Control, NPK1, NPK2, NPK3, NPK4) on the yield and stability of winter wheat following alfalfa. In total, 19 seasons were analysed. The weather changed considerably at the experimental site. Significant increases in minimal, mean, and maximal temperatures were dated to the period 1987–1988, while precipitation remained the same to this day (insignificantly increasing trend by 0.5 mm per annum). Rising temperatures in November, May, and July positively affected wheat grain yield, especially in treatments with higher N doses. No relationship between yield and precipitation was recorded. Highest inter-annual yield variability was recorded in Control and NPK4 treatments. Although minerally fertilized treatments provided slightly higher yields, the difference between Control and NPK treatments was insignificant. According to the linear-plateau response model, the recommended dose of 44 kg ha^−1^ N corresponds with yield of 7.4 t ha^−1^, while Control provides an average yield of 6.8 t ha^−1^. The application of higher doses did not lead to significant grain yield increase. Alfalfa as a preceding crop reduces the need of N fertilization and contributes to sustainable conventional agriculture, however, its share in crop rotations is decreasing both in the Czech Republic and in Europe.

## 1. Introduction

Wheat is the most important cereal crop in the Czech Republic, covering approximately 32% of the total sown area (2000–2022), and accounts for 57% of the total area under cereal cultivation [1]. Productivity of wheat depends on a wide range of factors, such as tillage management [2], variety selection [3], and, mainly, fertilization and weather.

The most important nutrient for wheat is nitrogen (N), significantly affecting its yield and quality [4,5,6,7,8]. The availability of N fertilizers, together with socio-economic, technological, and genetic development, led to the advance of crop production in the 20th century [9]. As a consequence, consumption of N fertilizers increased worldwide [10], in China [11], USA [12], and Europe [13], increasing food and feedstuff production and mankind’s development. However, over time, it has become clear that the application of mineral fertilizers also has undesirable side-effects, especially on the environment [14,15,16,17,18]. Application of excessive amounts of mineral N also negatively affects yield and quality of arable crops [19,20]. Especially in the case of wheat, the risk of reduced yield, quality, and harvestability increases with unnecessary high rates of mineral N due to lodging and changes in wheat metabolism [21,22]. Finally, unreasonably high amounts of mineral N are also financially unprofitable. Especially nowadays, when prices of N fertilizers have increased in the Czech Republic in 2022, due to the current energy crisis, by 84–150% compared to 2011 (depending on the type of fertilizer). For these reasons, mineral fertilizer rates ought to be optimised—to achieve reasonable yields with the most appropriate rate of applied fertilizers. For optimisation, experiments with different, increasing fertilizer rates are usually used. Based on the yields, recommendations are calculated using various statistical methods (ANOVA for example) and response models, such as quadratic, quadratic—plateau—logistic and so on [23]. These experiments usually last from one to several years [7,24,25,26] and provide useful information, especially about specific crop variety used in the trial, but cover short times from the point of weather variability—the duration of experiments may exclude seasons with abnormal conditions, such as intensive dry episodes, which are increasingly common in the Czech Republic and worldwide [27,28,29,30]. The increased frequency of abnormal weather conditions is caused by the climate change. Definition of climate change is problematic [31] but can be generally characterized as long-term climate shifts in rainfall and temperature, directly affecting inter-annual weather variation (higher incidence of extraordinary conditions), and thus agriculture production [32,33,34,35,36,37,38]. Effects of weather and inter-annual variation and fertilization on yield and its stability can be studied through long-term trials. These trials cover wide ranges of weather conditions, both normal and abnormal, thus their explanatory value of optimization is higher than in short-term trials. They also allow us to analyse how long-term conditions in particular months affect crop production and the relationship between weather, fertilization rate, and yield, in other words, yield stability. For example, barley production in long-term trials in Germany was mainly affected by weather conditions (55%), while fertilization participated by only 11% on yield formation. Rainfall from April to July was positively correlated with grain yield, while March precipitation and April temperature showed a negative correlation when high mineral N rates were applied [39]. According to Addy et al. [40], who analysed long-term trials in Rothamsted, England, wheat grain yield was sensitive to the N rate and mean temperature in November, April, and May, and to rainfall in October, February, and June, while barley yields were correlated to mean temperatures in February and June, and rainfall in April to July and September. In the United States, between 1950 and 2016, inadequate rainfall was the primary cause of wheat grain yield variation, when limited rainfall in April and May reduced yields in Oklahoma, while May and June–July rainfall were dominant factors explaining yield variation in Kansas and North Dakota, respectively [41]. However, long-term trials also have their drawbacks. In order to keep up with the times, it is necessary to alternate growing crop varieties. Different varieties may respond slightly differently to fertilization and weather conditions. This reduces the accuracy of the results, which, in the long run, does not relate to a particular or specific crop variety but, instead, to a plant species.

Negative impacts of inter-annual weather variability can be mitigated by appropriate form and rate of fertilizers and by suitable crop rotation. Mineral N and diverse crop rotations are the most important factors increasing yield stability, while minerally unfertilized fields and fields treated only with organic manures are more prone to instability [39,42,43]. Composition of crop rotation and the choice of preceding crop also significantly influences the productivity of arable crops [43,44,45,46]. Wheat appears to be the least suitable preceding crop for itself. With each successive season, when wheat follows wheat, yields decrease and even the application of N fertilizers cannot break the trend [47]. On the other hand, legumes seem to be an optimal choice for wheat [25,48,49,50]. As legumes are able to fix the atmospheric N due the symbiotic association with microorganisms, they make N available to crops and soil microbiota, improve soil fertility, and significantly reduce the need of N fertilizers for the following crop, saving the environment and the farmer’s finances [51,52]. Unfortunately, legumes production in Europe is declining at the expense of wheat and rape [52]. The same situation is visible in the Czech Republic, where decline of fodder crops follows decreasing livestock production [1,53].

In this paper, we evaluated weather development (1955–2022) and wheat grain yield from 19 seasons, when wheat followed alfalfa in the crop rotation, and five fertilizer treatments of the long-term trial, established in Prague in 1955, with the aim to analyse: (i) development of weather conditions at the site (H_0_: weather is not changing; H_A_: weather is changing); (ii) relationship among weather, N rate, and grain yield (H_0_: no relationship exists; H_A_: relationship between the factors exists); (iii) effect of N rate on yield stability (H_0_: different rates provide equal stability; H_A_: stability is sorted); (iv) how different N rates affect winter wheat grain yield (H_0_: increasing N rates provide comparable yield; H_A_: yield among treatments is different); and (v) the reasonable dose of mineral N (rate optimisation).

## 2. Results

### 2.1. Weather Development (1954–2022)

**Figure 1:**
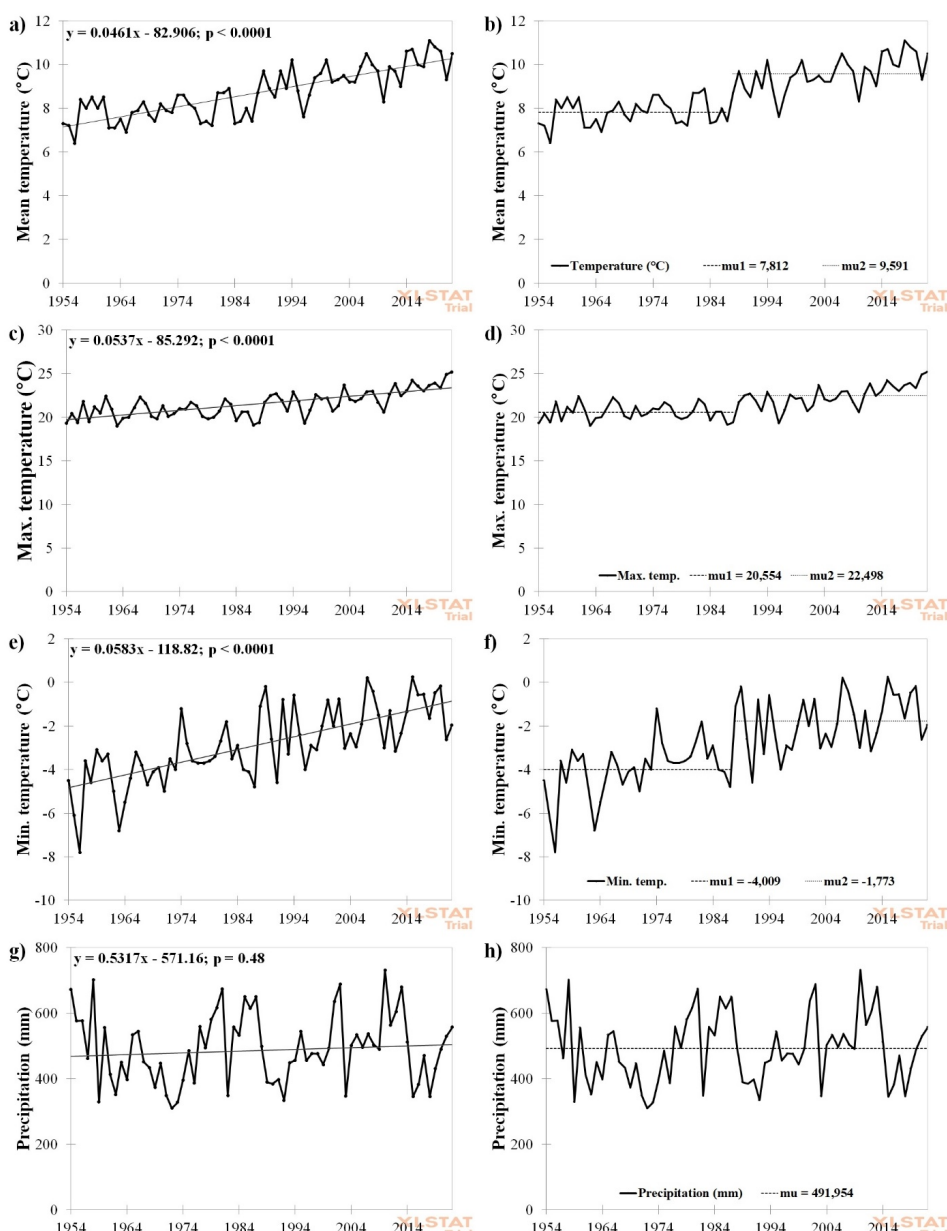
Development and year of change in mean (**a**,**b**), max. (**c**,**d**), min. (**e**,**f**) temperatures and (**g**,**h**) precipitation. The x values—the magnitude (annual change; °C for temperature; mm for precipitation); p—significance level from Mann—Kendall test; mu1—mean temperature before the year of change; mu2—mean temperature after the change in year.

We observed significant temperature changes at the trial site since its founding. The mean, max., and min. temperatures continually increased. The significant temperature change was dated to 1987 (mean and min. temperatures) and 1988 (max. temperature). Before these years, the mean temperature was 7.8 °C, while the following period was characterised by a mean temperature of 9.6 °C. The average maximal temperature increased from 20.6 °C to 22.5 °C and minimal temperature from −4.0 °C to −1.8 °C between these periods. On the other hand, precipitation has not changed significantly over time. The evolution of individual temperatures and precipitation is shown in Figure 1. Generally, the climate at the trial site is annually warming by 0.04–0.05 °C, while precipitation remains unchanged (insignificant annual rise by 0.5 mm); thus, crops have had to deal with significantly higher temperatures since 1987–1988, with precipitation equal to times before the temperature rise.

### 2.2. Relationship between Weather and Grain Yield

**Figure 2:**
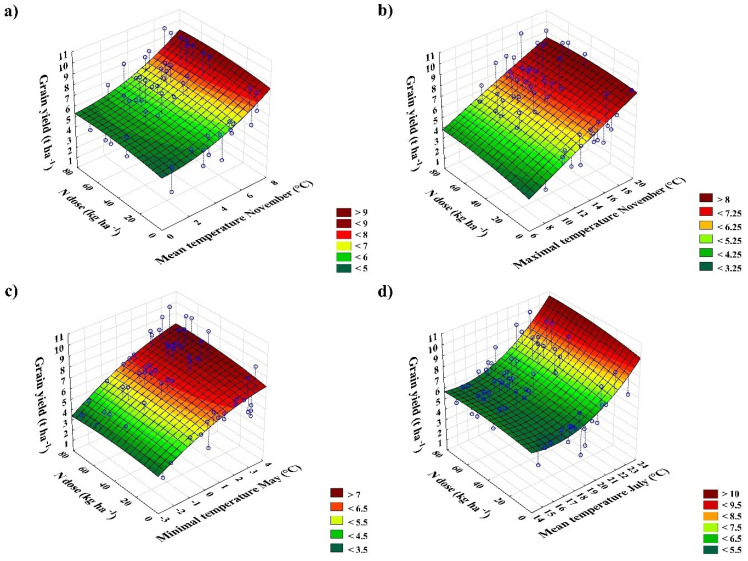
Relationship among temperatures (°C), N dose (kg ha^−1^) and grain yield (t ha^−1^); (**a**) mean temperature in November, (**b**) max. temperature in November, (**c**) min. temperature in May, (**d**) mean temperature in July. The numerical and colour scales in the legend represent the temperature gradation.

The correlation analysis between mean, max., min. temperatures and grain yield confirmed four statistically significant relationships: (i) mean temperature in November (r = +0.5, Figure 2a), (ii) max. temperature in November (r = +0.5, Figure 2b), (iii) min. temperature in May (r = +0.6, Figure 2c), and (iv) mean temperature in July (r = +0.5, Figure 2d). Relationship between grain yield and precipitation was insignificant. Generally speaking, warmer conditions in November, May, and July increase the chances of higher yields. This chance is enhanced by increasing mineral N rates; thus, more intensively fertilized wheat crops benefit more from the warmer weather. This section may be divided by subheadings. It should provide a concise and precise description of the experimental results, their interpretation, as well as the experimental conclusions that can be drawn.

### 2.3. Grain Yield Stability

The highest yield stability was provided by NPK3 treatment (rank 1), followed by NPK 2 (rank 2), NPK1 (rank 3), NPK4 (rank 4), and Control (rank 5) treatments. This means that the unfertilized Control provided the least stable yields, was the most vulnerable to weather effects, and yields showed a high degree of inter-annual variability. Application of mineral fertilizers acted as a stabilizing element that reduced the degree of yield inter-annual variability. In terms of stability, the highest dose of mineral N ranked second to last, while lower doses provided higher stability.

### 2.4. Effect of Fertilization on Grain Yield and N Rate Optimization

According to the Kruskal–Wallis test results, increasing doses of mineral N had an insignificant effect on the wheat grain yield of both the LS (d.f. = 4, *p* = 0.692) and SS (d.f. = 4, *p* = 0.274) wheat varieties (application of different fertilizer doses resulted in comparable grain yields in LS and SS varieties). The LS varieties gave a mean grain yield of 5.4 t ha^−1^, while the SS varieties provided an average yield 1.9 t ha^−1^ higher (Table 1). According to the Mann–Whitney test, the difference between the mean grain yield of the LS and SS varieties was significant (*p* < 0.0001). The same applied to the comparison of different fertilizer treatments (Control LS vs. Control SS, …, Table 1).

**Figure 3:**
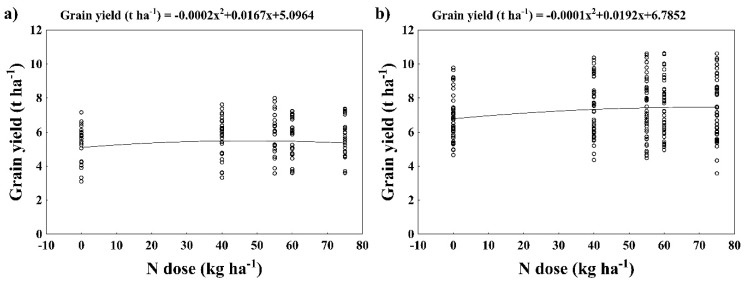
Grain yield (t ha^−1^) as affected by N dose (kg ha^−1^). (**a**) LS wheat varieties (1957–1980, 8 seasons), (**b**) SS wheat varieties (1988–2016, 11 seasons). Data (black circles) are interleaved with quadratic model (black line).

According to the quadratic model, the maximal mean grain yield of LS varieties occurred at the rate of 42 kg ha^−1^ N, corresponding with the yield of 5.4 t ha^−1^ (Figure 3a). For SS varieties, local maximum of the quadratic function was found at the rate of 70 kg ha^−1^ N, corresponding with the yield of 7.5 t ha^−1^ (Figure 3b).

**Figure 4:**
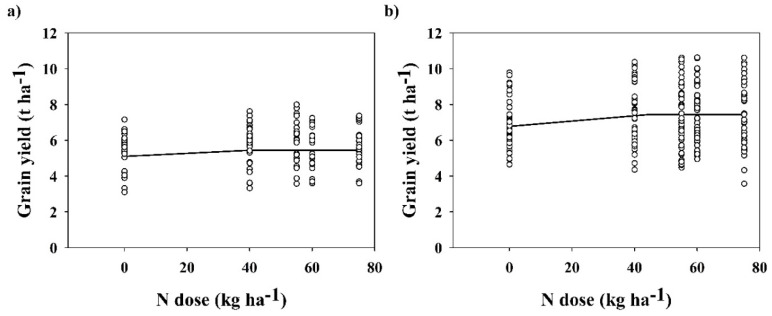
Grain yield (t ha^−1^) as affected by N dose (kg ha^−1^). (**a**) LS wheat varieties (1957–1980, 8 seasons), (**b**) SS wheat varieties (1988–2016, 11 seasons). Data (black circles) are interleaved with linear-plateau model (black line).

As the linear-plateau model revealed, the shoulder point of LP wheat varieties occurred at the rate of 40 kg ha^−1^, corresponding with the yield of 5.4 t ha^−1^ (Figure 4a). That was the same yield established by the quadratic model, but with the reduction of 2 kg ha^−1^ N. Combining the quadratic and linear-plateau models would not produce a significant change in yields, thus we could use the result of the linear-plateau model as a reasonable dose of N recommendation. In SS varieties, the shoulder point increased (compared with LP varieties) to 44 kg ha^−1^, corresponding with the yield of 7.4 t ha^−1^ (Figure 4b). As in the previous case, the combination of quadratic and linear-plateau models would not produce a significant change in yield (the difference is 100 kg ha^−1^; 7.4 t ha^−1^ vs. 7.5 t ha^−1^ with the reduction of 26 kg ha^−1^ N); thus, the result of the linear-plateau model could be considered as a recommendation for a reasonable dose.

## 3. Discussion

### 3.1. Weather

Weather conditions (temperature) at the long-term experiment site changed significantly over time. The temperature is increasing and gradually warming. This trend is global, recorded by other researchers in the Czech Republic [54,55], Poland [56], Germany [57], Austria [58], France [59], Europe [60], the USA [61], and Asia [62]. On the other hand, precipitation is relatively stable, with insignificantly increasing linear trends in the Czech Republic [63] or in the neighbouring Poland [64]. The fact that seasonal and monthly rainfall distribution changes is also important [64]. As a result, warmer weather leads to greater evaporation and surface desiccation. Heat waves and droughts are predicted to occur more frequently [65,66], and raising air temperature also increases the air’s water holding capacity and the water vapor in atmosphere, thus rainstorms are more intensive, even in areas with decreasing precipitation [67]. All these contexts have a significant impacts on crop production, resulting in yield stagnation or predicted reduction [68,69,70]. On the other hand, some authors suggested that current weather development remains or will be beneficial for wheat [54,71,72], especially in higher elevations [73]. We have analysed these consequences because an interesting situation was revealed in our study. The introduction of SS wheat varieties into the trial in 1988 significantly increased grain yield (Table 1). These varieties with a shorter stature were resistant to lodging and reached anthesis earlier than old varieties with a longer stature but required more intensive agronomy to utilize their yield potential (higher doses of N fertilizers and more intense plant protection) [74]. Similar results with a significant difference between LS and SS wheat varieties were recorded by Hejcman et al. [75,76,77], who analysed three long-term trials running under different soil–climate conditions in the Czech Republic (weather assessment weas not included). However, the same period (1987–1988) also included the beginning of a significant weather change at the site of our trial (see Section 2.1.). From these years onwards, a period characterised by higher temperatures and comparable precipitation to the previous period began. The coincidence of such significant factors prevents a clear answer to the question of what is behind the significant increase in grain yields in our trial. We assume that both factors contributed to the higher wheat yields. First, the increase in yields associated with the introduction of SS varieties is a well-known fact [74,78,79]. Secondly, from the weather analysis, we also know that warmer weather in autumn, spring, and summer is positively correlated with yields (Figure 2). This is due to higher chances for seedling emergence and a longer time window for plant development before winter (better chances for overwintering). Higher temperatures in May promote wheat growth and development after winter and reduces the risk of spring frosts (occurring from the beginning of April to the end of May), and higher summer temperatures speed up ripening and reduce risk of root and stem lodging.

### 3.2. Yield Stability

The lowest yield stability was recorded in Control treatments. It means that unfertilized crops (without any N fertilizers) showed the highest inter-annual grain yield variability and heavily relied on weather course and nutrients available from soil. Similar results were found by [39,42,43,80]. This is quite important information for organic farming, where mineral fertilizers are restricted, thus, other stabilization mechanisms, such as diversified crop rotations or organic manure application [43,81], ought to be incorporated. Application of mineral P+K fertilizers (without N) provided stability comparable to unfertilized treatments [42], thus mineral N seems to be a principal factor for yield stability, significantly reducing inter-annual yield variability. This is in agreement with our results, as highest stability was recorded in the NPK3 treatment, followed by the NPK2 and NPK1 treatments. The NPK4 treatment ranked second to last (before Control), showing lower stability in comparison with the NPK1–NPK3 treatments. Similar results were recorded by Macholdt et al. [43], where higher stability provided 70 kg ha^−1^ N in comparison with 140 kg ha^−1^ N. Cereals had a specific response to increasing doses of mineral N, they responded concavely. After reaching a local maximum, yields decreased with increasing N doses [82,83]. Therefore, unnecessarily high doses of mineral N not only endanger the environment, they are also unprofitable in terms of efficiency, finances, and stability.

### 3.3. Effect of Fertilization on Wheat Grain Yield

Nitrogen is the most important nutrient for arable crops, including wheat [8]. In the past, N fertilization was considered a secondary issue as the natural supply from crop rotations (including legumes), post-harvest residues incorporation, and organic manures was sufficient [84]. During the 20th century, however, the situation changed. While the demand for mineral fertilizers, especially N, is increasing, in some countries (such as the Czech Republic), crop diversity and livestock numbers are being reduced [85], including legumes [52]. As mentioned above, legumes are able to increase soil fertility and provide N [51,52], replacing part of the mineral fertilizers applied to the following crop. This can be from 37 kg ha^−1^ [86], 51 to 62 kg ha^−1^ [87], and up to 76 kg ha^−1^ for wheat following alfalfa and maize [49]. In some cases, N fertilization was even not necessary for wheat following alfalfa [50]. Integrating alfalfa into crop rotation enables shorter cropping intervals of sugar beet without yield loss [46] and reduction in N fertilizers (up to 100 kg ha^−1^) to maize growing second year on the field (following alfalfa and maize in crop rotation), with small or no economic penalties [88]. In our case, no differences among fertilizer treatments were recorded in both LS and SS wheat varieties (see Section 2.4). Even the unfertilized Control treatment offered yields statistically comparable to NPK treatments, and the average grain yield of 6.8 t ha^−1^ (Table 1) far exceeds the average grain yield of the Czech Republic in 2017–2021, which was 5.85 t ha^−1^ [89]. This implies that a source other than applied N fertiliser contributed to yield production and caused insignificant differences between fertilizer treatments. According to the above-mentioned papers, we assume that this insignificance was caused by alfalfa. In other words, alfalfa was able to provide sufficient amount of N to wheat, resulting in satisfactory grain yield even in unfertilized Control treatments (5.1 t ha^−1^ and 6.8 t ha^−1^ in LS and SS varieties, respectively). Slightly higher grain yields could be harvested after application of 40–42 kg ha^−1^ N in the case of LS varieties and 44 kg ha^−1^ N regarding the SS wheat varieties. The increase was 300–400 kg ha^−1^ (LS) and 600–700 kg ha^−1^ (SS), but this increase was evaluated as insignificant in our evaluation. The application of higher doses of mineral N is not necessary under soil–climate conditions comparable to the conditions of our trial. In comparison with the previous study [90] that evaluated the identical long-term trial in Prague, but with potatoes as preceding crop to wheat, the recommended dose of mineral N for wheat was 87 kg ha^−1^ N, corresponding with the yield of 6.9–7.0 t ha^−1^ (SS varieties). Thus, the difference between potatoes and alfalfa as a preceding crop was an increase in average yield of 300 to 400 kg ha^−1^ in favour of alfalfa, at an N rate 43 kg ha^−1^ or lower. The beneficial effect of alfalfa as a preceding crop, which positively affected the yield of the following crop, was also demonstrated by the comparison of potatoes and alfalfa in the unfertilised Control treatment. According to [90], the average yield of winter wheat following potatoes was 3.8 (LS) and 4.7 (SS) t ha^−1^. For alfalfa, the average yields were 5.1 (LS) and 6.8 (SS) t ha^−1^, a difference of 1.3 and 2.1 t ha^−1^, respectively. This supported our assumption of the positive effect of alfalfa as a preceding crop, which improved soil properties and nutrient mineralization [91], allowed a reduction in mineral N applied, and even achieved higher yields when compared with potatoes as a preceding crop.

## 4. Materials and Methods

### 4.1. Trial Description

The long-term field trial was established on the north-western edge of Prague (50°05′15″ N, 14°17′28″ E), the Czech Republic, Central Europe in 1955. It was located in a warm-summer continental climate area (Köppen–Geiger climate classification—Dfb [92]). The long-term mean temperature was 8.7 °C, and precipitation was 492 mm (1954–2022, Crop Research Institute meteorological station). Elevation of the site was 370 m above sea level. Soil type was Orthic Luvisol [93], formed by diluvial sediments mixed with loess, and the topsoil depth was approximately 0.3 m.

Five fertilizer treatments were evaluated in this paper: (i) Control (unfertilized since trial establishment); (ii) NPK1 (40, 21, 80 kg ha^−1^ NPK); (iii) NPK2 (55, 26, 100 kg ha^−1^ NPK); NPK3 (60, 21, 80 kg ha^−1^ NPK); and NPK4 (75, 26, 100 kg ha^−1^ NPK). Doses were expressed as concentrations (kg ha^−1^) of pure elements. Mineral N was applied as calcium ammonium nitrate (27% N), P as super phosphate (8.3% P), and K as potassium chloride (49.8% K). Mineral P and K fertilizers were applied in autumn, incorporated by moderate tillage (single dose). Mineral N was applied in spring (single dose). Application of mineral fertilizers was carried out by hand spreading. Each fertilizer treatment was replicated four times in a completely randomized block design. The size of individual plot was 12 × 12 m, but only central area (5 × 5 m) was used for collection of scientific data (the edge effect elimination).

Wheat grain yield results analysed in this paper were collected from three fields with the same crop rotation consisting of alfalfa, winter wheat, sugar beet, spring barley, potatoes, winter wheat, sugar beet, and spring barley (45% cereals, 33% root crops, 22% legumes). Winter wheat, which followed alfalfa in the crop rotation, was harvested over 19 seasons (1957, 1959, 1966, 1967, 1968, 1977, 1979, 1980, 1988, 1989, 1995, 1997, 1998, 2004, 2006, 2007, 2013, 2015, and 2016). Long-strawed (LS) wheat varieties were grown between 1957 and 1980, while short-strawed (SS) wheat varieties were grown between 1988 and 2016. As different LS and SS wheat varieties were grown during the experiment, the effect of fertilizer treatment on grain yield was focused on two groups (LS and SS varieties). This step was taken because weather conditions were different each year and different varieties could react differently. Nevertheless, we believe that the predictive value of the long-term time series provided a sufficient characterisation of wheat and its reaction to fertilizers as a whole. Sowing was usually performed in October to a depth of 0.03–0.05 m and harvesting in late July or early August, according to maturity. The row distance was 0.125 m.

### 4.2. Weather Analysis

The development of weather was analysed by Mann–Kendall test and Sen’s estimator of slope, together with the test of homogeneity, using the XLStat software (Lumivero, Burlington, MA, USA). To analyse the correlation between weather parameters and wheat grain yield, the minimum, mean, and maximum temperature and precipitation of a given month (October–August; long-term average values) and grain yield were considered.

### 4.3. Data Analyses

The data normality (grain yield results) was checked by a Shapiro–Wilk test [94]. The effect of fertilizer treatment on grain yield of LS and SS wheat varieties was analysed by Kruskal–Wallis one-way ANOVA (comparison of Control, NPK1, NPK2, NPK3, and NPK4 treatment within the LS and SS varieties). The difference between mean grain yield of LS and SS varieties was performed using the Mann–Whitney test (comparison of mean grain yields between LS and SS varieties). These analyses were calculated, as well as graphical outputs, using the Statistica 14.0 (TIBCO Software, Palo Alto, CA, USA). The response models for N rate optimization (quadratic and linear plateau) were calculated using SigmaPlot 14.5 (Systat Software Inc., San Jose, CA, USA). Yield stability was calculated by the Kang’s rank-sum statistic [95], using the StabilitySoft [96]. The Kang’s rank-sum analysis uses yields and Shukla’s stability variance analysis as selection criteria. The Kang’s rank-sum statistic gave a weight of one to both yield and stability statistics to identify high-yielding and stable treatments. The fertilizer treatment with the highest yield and lower value of Shukla’s stability variance were assigned a rank of one. Then, the ranks of yield and stability variance were added for each treatment, and the treatment with the lowest rank-sum was the most desirable [96].

## 5. Conclusions

(i) Weather was changing significantly at the trial site. Mean, maximal, and minimal temperatures annually rose by 0.05 °C, 0.05 °C, and 0.06 °C, respectively. The change in temperatures was dated to the period 1987–1988. Before these years, the mean temperature was 7.8 °C, while the following period was characterised by a mean temperature of 9.6 °C. The average maximal temperature increased from 20.6 °C to 22.5 °C and the minimal temperature from −4.0 °C to −1.8 °C between these time periods. On the other hand, precipitation remained unchanged, with an insignificantly increasing trend of 0.5 mm per annum, thus, from these years onwards, crops had to cope with higher temperatures with generally the same amount of water as before these years.
(ii) A positive relationship between temperatures in November, May, and July and grain yield was recorded, especially in treatments with higher doses of nutrients. Warmer temperatures in November increased chances for seedling emergence and crops had better conditions for proper development before winter with, consequently, better prospects for overwintering. Higher temperatures in May promoted development after winter and reduced the risk of spring frosts, while warmer temperatures in July sped up ripening and reduced the risk of root and stem lodging.
(iii) Lowest inter-annual yield variability was recorded in NPK3 treatment, followed by lower doses of nutrients (NPK2 and NPK1), while lower stability was recorded in NPK4 and Control treatments. Without proper fertilization, crops are more vulnerable to weather fluctuations, while proper nutrition increases environmental adaptability. On the other hand, too many nutrients can be counter-productive under unsuitable weather conditions, especially in the case of wheat (concave progress of quadratic response model).
(iv) Wheat following alfalfa in the crop rotation required lesser doses of mineral fertilizers and offered higher yields in comparison with other preceding crops (such as potatoes). In comparison with previous studies that evaluated wheat yields in the same trial, but with potatoes as preceding crop [90], the mean grain yield of SS varieties following alfalfa was 300–400 kg ha^−1^ higher, with N rates being 43 kg ha^−1^ lower. The average wheat grain yields in the unfertilized Control treatment were 3.8 (LS) and 4.7 (SS) t ha^−1^ after potatoes [90], while 5.1 (LS) and 6.8 (SS) t ha^−1^ after alfalfa. This yield from the SS Control treatment (6.8 t ha^−1^) even exceeded the average wheat grain yield harvested in the Czech Republic between 2017 and 2021 (5.85 t ha^−1^). The application of mineral fertilizers slightly increased wheat grain yield (by 300–400 in LS varieties and 600–700 kg ha^−1^ in SS varieties), but the difference between the Control and NPK treatments was insignificant for both groups (LS and SS varieties). The application of 44 kg ha^−1^ N led to an average yield of 7.4 t ha^−1^, representing a 600 kg ha^−1^ (SS varieties) increase in comparison with the unfertilized Control. The application of higher doses did not lead to significant grain yield increases.

## Figures and Tables

**Table 1:** Minimal, maximal and mean grain yield (t ha^−1^) of LS and SS wheat varieties as affected by fertilizer treatment. SD = standard deviation. LS and SS mean grain yields, followed by the same capital letter, are not significantly different (α < 0.05).

	LS Varieties				SS Varieties			
	Min.	Max.	Mean	SD	Min.	Max.	Mean	SD
Control	3.1	7.2	5.1 ^A^	1.2	4.7	9.8	6.8 ^B^	1.3
NPK1	3.3	7.6	5.5 ^A^	1.4	4.4	10.4	7.4 ^B^	1.6
NPK2	3.6	8.0	5.5 ^A^	1.4	4.8	10.6	7.4 ^B^	1.8
NPK3	3.6	7.2	5.5 ^A^	1.3	5.0	10.6	7.5 ^B^	1.6
NPK4	3.6	7.4	5.4 ^A^	1.3	3.6	10.6	7.5 ^B^	1.8
Mean	3.4	7.5	5.4 ^A^		4.5	10.4	7.3 ^B^	

## Data Availability

Not applicable.
